# Application of artificial intelligence chatbots, including ChatGPT, in education, scholarly work, programming, and content generation and its prospects: a narrative review

**DOI:** 10.3352/jeehp.2023.20.38

**Published:** 2023-12-27

**Authors:** Tae Won Kim

**Affiliations:** AI‧Future Strategy Center, National Information Society Agency of Korea, Daegu, Korea; Hallym University, Korea

**Keywords:** Artificial intelligence, Literacy, Reproducibility of results, Search engine, Writing

## Abstract

This study aims to explore ChatGPT’s (GPT-3.5 version) functionalities, including reinforcement learning, diverse applications, and limitations. ChatGPT is an artificial intelligence (AI) chatbot powered by OpenAI’s Generative Pre-trained Transformer (GPT) model. The chatbot’s applications span education, programming, content generation, and more, demonstrating its versatility. ChatGPT can improve education by creating assignments and offering personalized feedback, as shown by its notable performance in medical exams and the United States Medical Licensing Exam. However, concerns include plagiarism, reliability, and educational disparities. It aids in various research tasks, from design to writing, and has shown proficiency in summarizing and suggesting titles. Its use in scientific writing and language translation is promising, but professional oversight is needed for accuracy and originality. It assists in programming tasks like writing code, debugging, and guiding installation and updates. It offers diverse applications, from cheering up individuals to generating creative content like essays, news articles, and business plans. Unlike search engines, ChatGPT provides interactive, generative responses and understands context, making it more akin to human conversation, in contrast to conventional search engines’ keyword-based, non-interactive nature. ChatGPT has limitations, such as potential bias, dependence on outdated data, and revenue generation challenges. Nonetheless, ChatGPT is considered to be a transformative AI tool poised to redefine the future of generative technology. In conclusion, advancements in AI, such as ChatGPT, are altering how knowledge is acquired and applied, marking a shift from search engines to creativity engines. This transformation highlights the increasing importance of AI literacy and the ability to effectively utilize AI in various domains of life.

## Graphical abstract

[Fig f4-jeehp-20-38]

## Introduction

### Background

Generative Pre-trained Transformer (GPT) is a natural language generation model developed by OpenAI. It learns to predict the next word in a given text, generating meaningful text that resembles human-written content [1]. The performance of GPT is contingent upon the number of parameters it employs. GPT-3.5, released on November 30, 2022, utilizes approximately 1,500 times more parameters (175 billion) than GPT-1 (117 million), which was first released in 2018.

ChatGPT is a chatbot built upon GPT-3.5. It comprehends sentences entered by users, generates relevant responses, and communicates with users utilizing everyday language, akin to human-to-human conversation. While GPT-3.5 has parameters equivalent to GPT-3 (175 billion), engendering negligible performance discrepancies, it was optimized for conversation by applying reinforcement learning from human feedback (RLHF). Understanding ChatGPT, which boasts a diverse spectrum of applications encompassing education, research, and programming, and discerning its domains of applicability and limitations will be instrumental in effectively harnessing this artificial intelligence (AI) chatbot.

### Objectives

This review aims to present perspectives on topics that merit attention by examining various use cases and limitations of ChatGPT. Specifically, it explains ChatGPT’s reinforcement learning and describes its use cases, limitations, and perspectives. This information can be helpful when utilizing AI-based natural language processing models, such as ChatGPT, in education and academic fields. It will also aid in understanding new models as they emerge.

### Ethics statement

Since this was not a human population study, neither approval by the institutional review board nor obtainment of informed consent was required.

### Improvement of ChatGPT’s performance through reinforcement learning

In contrast to supervised or unsupervised learning, which occurs within a static framework where the data for learning are predetermined and unchanging, acquiring proficiency in a specific task through dynamic interactions with an uncertain environment constitutes reinforcement learning. This form of learning involves an agent situated within an environment, discerning the present state and ascertaining the action that maximizes potential rewards from a set of possible choices. The operational sequence of reinforcement learning is as follows: first, an agent, defined within an environment, observes the current state and executes an action; second, the agent adapts as the environmental state evolves, thereby altering the reward; third, the agent, guided by the modified rewards, learns to prioritize actions that yield more significant benefits, identifying the optimal course of action [2].

**Fig. 1.**
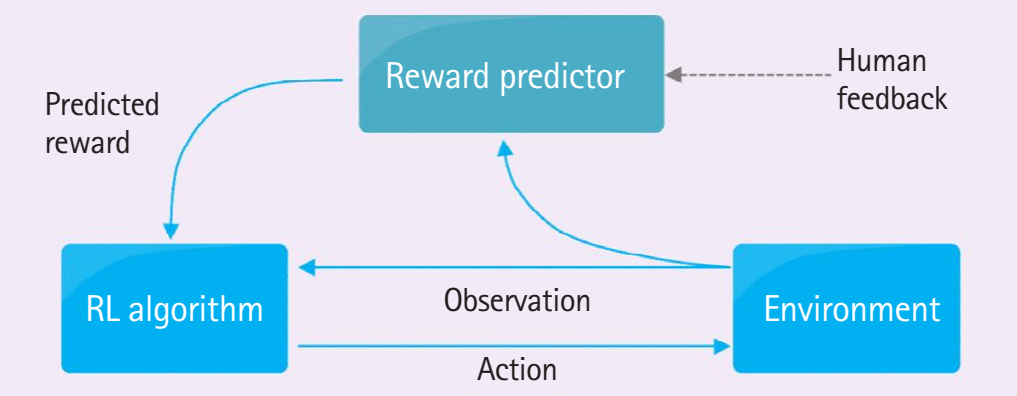
Diagram of the reinforcement learning (RL) process from human feedback. Modified from Learning from human preferences [Internet]. OpenAI; 2017 [cited 2023 Jan 26]. Available from: https://openai.com/blog/deep-reinforcement-learning-from-human-preferences/ [3].

Additionally, the RLHF technique has been incorporated into ChatGPT [3]. When augmented with human feedback, reinforcement learning captures human linguistic patterns and cultural nuances. Through the integration of RLHF, ChatGPT achieves a level of sentence construction so naturally human-like that it becomes virtually indistinguishable from human communication (Fig. 1) [3].

### Why are people enthusiastic about ChatGPT?

The genuine public interest in ChatGPT, an AI chatbot designed by OpenAI, is attributed to its multifaceted potential revealed following its release on November 30, 2022. Remarkably, the user base of ChatGPT reached 1 million within just 5 days of its launch and 2 million users within 2 weeks. This rapid adoption rate is significantly shorter than that of other primary services such as Netflix, which took 3.5 years; AirBnB, which took 2.5 years; and Facebook, which took 10 months to achieve the same milestone. This phenomenon represents an unprecedented achievement in the realm of AI services. In comparison, Copilot (https://github.com/features/copilot), an AI tool for coding assistance on GitHub, reached the 1 million user mark in six months, while DALL-E 2 (https://openai.com/dall-e-2/), OpenAI’s AI service for image creation, achieved this feat in approximately 2.5 months.

Upon its release, the explosive interest in ChatGPT can be credited to its robust performance across various applications, including the development of chatbots, essay composition, translation, content generation, and text summarization. Additionally, its capability to respond naturally to interactive queries has been a significant factor. The distinctions between ChatGPT and conventional search engines are delineated in Table 1.

ChatGPT is rapidly gaining a large and growing user base online, as the free service makes it easy for anyone interested in AI to try it out in chat and share their results on social media. In the future, OpenAI will offer a paid service called “ChatGPT Plus”[4]—a pilot subscription plan for ChatGPT, a conversational AI that can chat with users, answer follow-up questions and challenge incorrect assumptions.

## Use cases of ChatGPT in education, academia, programming, content generation, and implementing creative ideas

### Education

AI can create assignments and presentations and provide in-class discussions, questions, and personalized feedback. A major AI service like ChatGPT has the potential to be a great educational tool if utilized well. For example, in a parasitology exam, although ChatGPT’s knowledge and interpretation ability were not comparable to medical students in Korea, its performance was excellent, with a correct answer rate of 60.1% [5]. ChatGPT’s performance on the United States Medical Licensing Exam, consisting of Step 1, Step 2 CK, and Step 3, was at or near the cut score for all 3 examinations [6]. Of course, the best way to use AI is to use it as a complement rather than a replacement for human teachers. To appropriately adapt to an increasingly AI-enabled learning environment, students need digital training to recognize information sources, learn how to use automated AI models, and understand the limitations of automatic text.

Simultaneously, concerns exist about plagiarism and ghostwriting, the unreliability of results, copyright issues, educational gaps due to technological advancement, and decreased learning ability. Educators are concerned about students’ reliance on AI. Using ChatGPT to solve writing or computer coding assignments may hinder students’ learning, and New York City public schools have blocked access to ChatGPT on campus [7]. In response to these issues, OpenAI launched the AI Text Classifier—“a fine-tuned GPT model that predicts how likely it is that a piece of text was generated by AI from a variety of sources, such as ChatGPT” [8]. Teachers can use this tool to assess the students’ essays or reports. Furthermore, it can be used by scholarly journal editors to screen for AI-generated texts.

### Writing research articles and journal publishing

A variety of tasks can be performed from the research design stage to research writing using ChatGPT, including summarizing abstracts into a certain number of words, suggesting creative research titles, discussing experimental results, creating a research table of contents, recommending future research ideas, writing articles on specific topics, grammar correction of written content, and translation. Gao et al. [7] tested whether a scientist could detect 50 medical research abstracts produced by ChatGPT and found that a plagiarism checker found 0% of the abstracts, an AI output detector found 66%, and the scientist found 68%. In a review of the role of AI in drug discovery, Blanco-Gonzalez et al. [8] described the caveats of using ChatGPT for scientific writing. They said, “As an assistant to write scientific papers, ChatGPT has several advantages, including its capacity to generate and optimize text quickly, as well as to help users with several tasks, including organizing information or even connecting ideas in some cases. However, this tool is in no way ideal for generating new content. Our revision of the text generated by the AI, following our instructions, required major edits and corrections, including the replacement of nearly all the references since the ones provided by the software were clearly incorrect” [8]. A paper by O’Connor [9] on AI platforms in nursing education stated that the first five paragraphs were written by ChatGPT, and it also listed ChatGPT as a co-author. ChatGPT’s co-author inclusion later sparked a debate about ChatGPT’s authorship [10].

OpenAI said that “while ChatGPT’s ability to generate text in multiple languages may be useful for certain language-specific applications, such as content creation or language learning, it is not recommended to use ChatGPT for translation purposes. For accurate and reliable translations, it is recommended to use specialized translation tools and technologies” [11]. However, ChatGPT shows a significant level of performance compared to existing translators and can be used for teaching various foreign languages, including English, because it goes beyond simple translation and explains corrections and grammatical errors. It can also perform some level of translation and proofreading for academic publications. Later, more complete proofreading by a professional should be commissioned [12].

### Programming

ChatGPT can perform various programming tasks, such as writing simple program code, annotating, finding errors in the code (typos or undefined code), checking the reason for an error code, fixing the error code, guiding the user to install a program, and providing guidance on how to update a program. An example of programming with ChatGPT is shown in Supplement 1.

### Content creation

ChatGPT can go beyond simply answering users’ questions and create various content in creative forms, such as movie screenplays, novels, song lyrics, product flyers, advertising scripts, financial reports, contracts, proposals, and course curricula.

### Implementing creative ideas

ChatGPT can be used in endless ways depending on what one asks it, and in addition to the above uses, one can engage in the following kinds of creative tasks and documents. Tasks that can be delivered include the following: cheer up a depressed person, suggest a new product name, get podcast guest suggestions, make sure writing is free of racial bias, get dating tips, get gift ideas, make a workout plan, analyze text for sentiment, automatically comment on a restaurant blog, generate a list of questions for hiring, explain a complex concept (e.g., quantum physics to an elementary school student), and so forth.

The following types of text can be created: essays, news articles, blog posts, technical reports, business plans, funding applications, product manuals, user manuals, legal documents, contracts, resumes, cover letters, poems, novels, short stories, speeches, sermons, travel guides, textbooks, diaries, letters of recommendation, job ads, applications, brochures, and so on.

## Limitations of ChatGPT

### Functional limitations: Is ChatGPT fair and accurate?

ChatGPT can generate creative and professional answers not only to engineering problems, but also to literary, philosophical, and aesthetic problems, depending on how the user asks the question, and it provides results different from existing AI chatbot services. Through the application of RLHF, ChatGPT is recognized as a “game changer” that will change the search engine market with its smooth conversation and excellent answer performance.

**Fig. 2.**
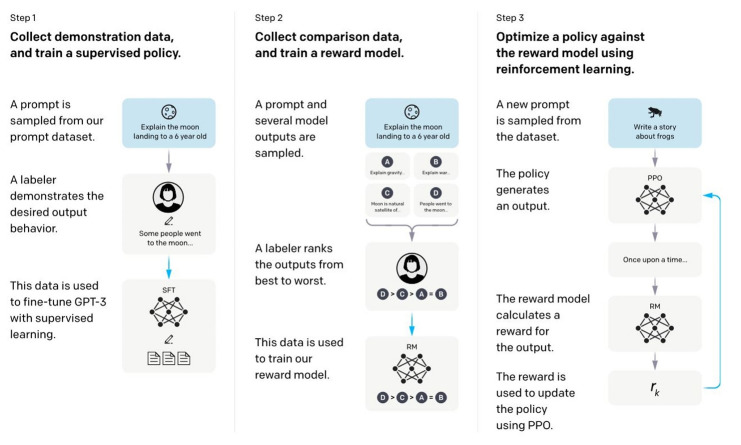
A diagram illustrating the 3 steps of the training method: (1) supervised fine-tuning (SFT), (2) reward model (RM) training, and (3) reinforcement learning via proximal policy optimization (PPO) on this reward model. Blue arrows indicate that this data is used to train one of the models. In Step 2, boxes A–D are samples from the models that get ranked by labelers. From Ouyang L, et al. Training language models to follow instructions with human feedback. arXiv [Preprint] 2022 Mar 4. https://doi.org/10.48550/arXiv.2203.02155 [13].

However, ChatGPT still has some imperfections and is subject to unintentional bias, which poses a risk. It is trained on data from before 2021 and may respond inaccurately to events after 2022. Because ChatGPT bases its answers on a large training data set, it has the potential to generate misinformation or biased content. Because it has more English data than other languages, it is more accurate for English queries. We are concerned that the application of RLHF may easily mimic human flaws and mistakes (Fig. 2) [13]. In particular, ChatGPT’s answers sometimes seem fairly logical, but it may give nonsensical answers (hallucination issue) or inaccurate answers, like seeing a hallucination that does not exist. Because of ChatGPT’s high rate of incorrect answers and generally plausible but poor-quality answers, the developer of the Q&A site Stack Overflow has banned ChatGPT-generated answers from being posted for the time being.

### Service limitations: Can ChatGPT generate sustainable revenue?

While ChatGPT has attracted 1 million users in a very short period compared to other services and is estimated to have over 500 million users, it is limited by the fact that it is a free service and does not have a significant revenue model. ChatGPT was able to reach 1 million users in a short period because it is a free service; anyone can quickly sign up for it, and users share and spread interesting results online due to the simplicity of the question method and excellent answer performance. The significance of ChatGPT lies not in its short-term user acquisition, but rather in the fact that it marks the beginning of generative AI. ChatGPT Plus, a paid service version, will be released in the future, and various customized services can be provided, so it will be necessary to develop a top-tier service that can ensure sustainability.

## Perspectives

### Can ChatGPT be a tool for innovation?

Technology is a tool that is neither good nor evil, and it is up to the user to decide how they want to use it. Therefore, for an AI service like ChatGPT to serve as a tool for innovation, it is necessary to always keep in mind the risks and side effects of using AI and make efforts to minimize them.

Overcoming limitations will help democratize AI: ChatGPT’s inaccurate and biased responses and errors in logically describing misinformation or nonsensical responses are expected to improve through repeated trial-and-error interactions. ChatGPT already uses the Moderation API, an AI-powered moderation system, to block discriminatory and hate speech. Thus, if a question is asked that is not appropriate, it will refuse to respond to discriminatory, offensive, or inappropriate questions, including racist, sexist, homophobic, transphobic, or otherwise discriminatory or hateful questions. ChatGPT is a language model, and its accuracy can be improved with further training and professional validation.

The future release of GPT-4 will accelerate the progress of super-scale AI: It has been predicted that GPT-4 will have 100 trillion parameters (571 times the number of GPT-3 parameters), equivalent to the number of human synapses (Note: the release of GPT-4 has already taken place on March 14, 2023).

### Less effort to gain knowledge

Knowledge refers to “a clear perception or understanding of an object gained through learning or practice,” and human knowledge is accumulated by learning from one’s own experiences and the experiences of others transmitted through various media. Therefore, learning is a set of processes that must be performed to build one’s knowledge, and knowledge cannot exist without learning or experience. The advent of search engines has dramatically reduced the time and cost of learning. AI services such as ChatGPT have revolutionized the definition of knowledge by eliminating the human learning process for knowledge acquisition.

### From the search engine era to the creativity engine era

**Fig. 3.**
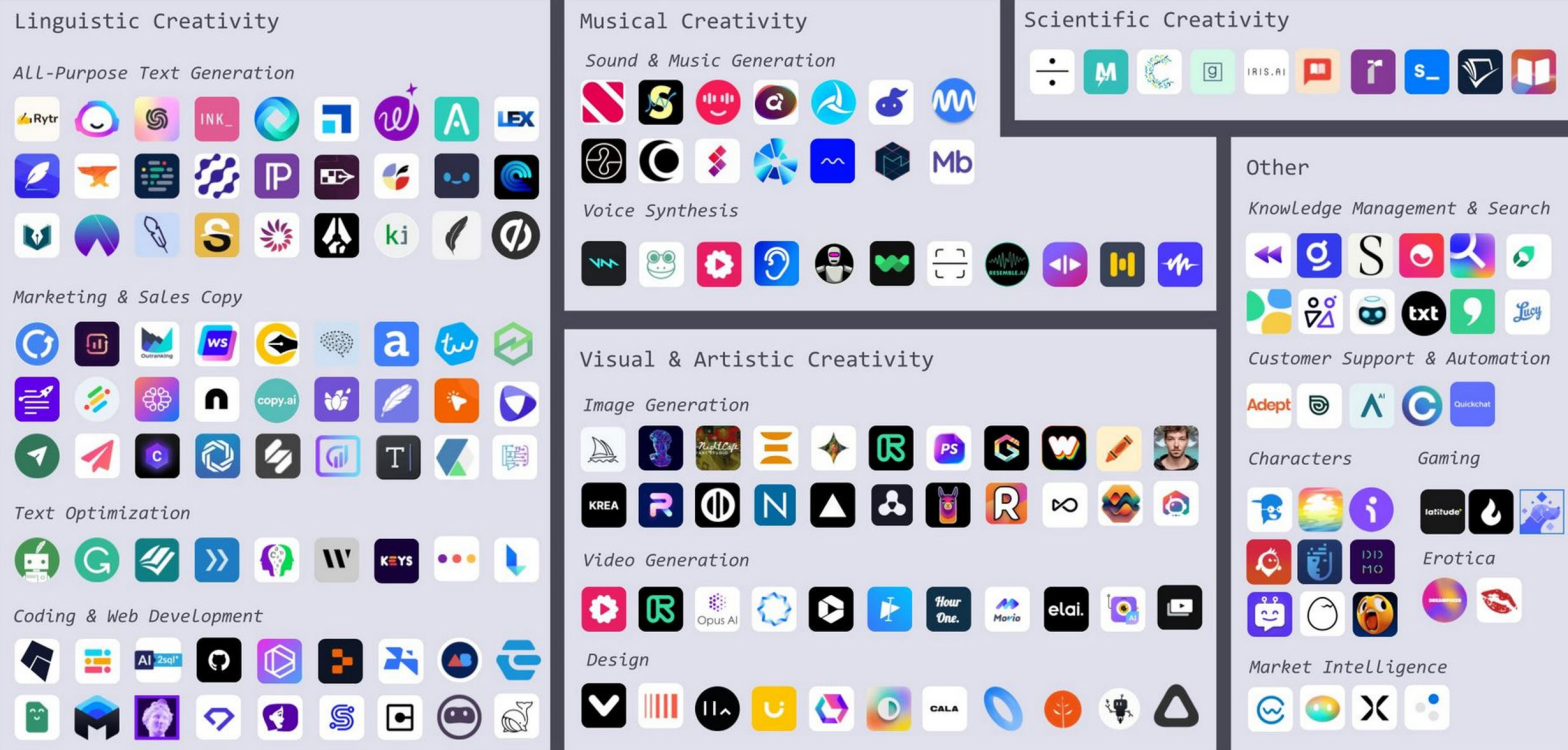
Artificial intelligence landscape. From Le Cunff AL. AI and I: the age of artificial creativity [Internet]. Ness Labs; c2022 [cited 2023 Jan 30]. Available from: https://nesslabs.com/artificial-creativity [14].

Until now, AI has been dominated by discriminative AI rather than generative AI due to technical issues. Discriminative models are suitable for analytical tasks such as image recognition based on supervised learning, while generative models are suitable for creative tasks such as image generation based on unsupervised learning. Generative AI has been limited by several factors over the years, including the difficulty of running models, the need for sophisticated workload balancing to manage computer resources and avoid bottlenecks, and the prohibitive cost of using cloud computing. However, with new technologies, more data, and cheaper computing power, it is now easier than ever to build generative AI. More than 180 AI tools exist for different types of creativity, including language, visual and art, audio and music, and science (Fig. 3) [14]. The emergence of powerful AI tools is expected to impact the lives of billions of workers, with widespread unemployment, some jobs being replaced, and others being expanded or reinvented in unexpected ways.

## Conclusion

The role of humans will change as technology advances. “The internet, with its easy access to information, robs us of the concentration and memory that comes from reading books, and while we may excel at information processing and decision-making, the brain’s habit of focusing on something and remembering it is declining” [15]. The advent of the automobile has made human mobility easier, the advent of computers has augmented our problem-solving abilities, and the advent of the internet may have reduced our attention span. Still, it has also created new value through connectivity. For example, with the advent of smartphones, it has become less important to remember a large number of phone numbers, and possession of information is not a power in itself. Nonetheless, the ability to find and connect scattered information is even more necessary. In the future, children will become AI natives who use AI to solve their questions. As AI services become more ubiquitous, “how well you handle AI” will become an essential competitive advantage for future generations, who will experience AI in every aspect of their lives and feel comfortable asking AI when they have questions.

## Figures and Tables

**Figure f4-jeehp-20-38:**
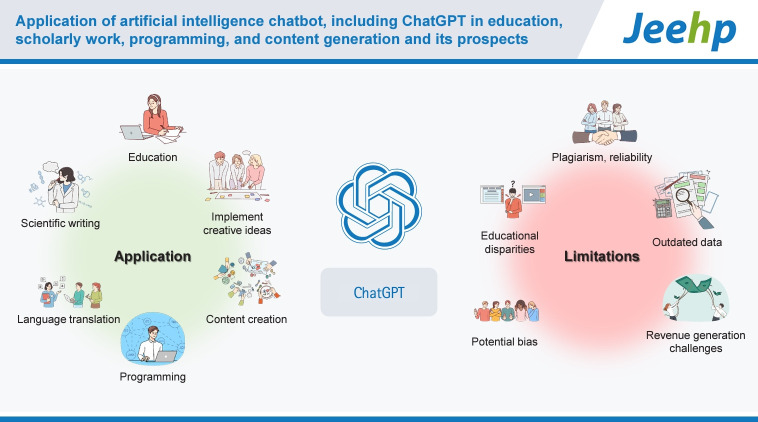


**Table 1.** Distinctions between ChatGPT and conventional search engines

	ChatGPT	Search engine
Artificial intelligence technology	Uses language modeling, an artificial intelligence technology, to generate answers to user questions	Provides information through keyword searching
Generative	Can generate new information about user questions, providing more generative answers than traditional search engines	Unable to create new information
Interaction	User-friendly interactions can help the user understand, as it can answer questions	No interaction with users, as it provides information through keyword searches
Questions and context comprehension	Natural language processing technology understands users’ questions to provide results that fit their intentions, remembers users’ previous questions, and answers flexibly considering their associations.	Provides information through keyword search rather than understanding and answering user questions, and provides information independently for each search^a)^

a) In recent years, search engines have also been evolving, applying natural language processing-based algorithms to analyze better and understand human language.

## Supplementary materials

Supplement 1. ChatGPT’s (2023 Jan 9 version) answer to the inquiry, “Could you generate code for machine learning in Python?” [cited 2023 Jan 23, 8:30 PM (Seoul time)].

Supplement 2. Audio recording of the abstract.
